# Supplementary figures and images for: Denoising diffusion probabilistic models for generation of realistic fully-annotated microscopy image datasets
Source: PLoS Comput Biol. 2024 Feb 20;20(2):e1011890. doi: 10.1371/journal.pcbi.1011890 (PMC10906858; doi:10.1371/journal.pcbi.1011890)

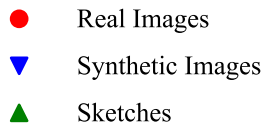

Supplement: S1 Fig — 2D feature representations obtained with t-SNE from the latent representation of an autoencoder for real 3D Arabidopsis thaliana image data [18] and corresponding synthetic data generated from sketches of manual annotations. Additionally, feature representations obtained for raw sketches serve as a reference. Since this data set contains large-scale image data, each image stack is partitioned into patches to reduce computational demand. During application, a feature representation for each single patch is obtained and averaged to derive an overall feature description of the entire image stack. Results indicate that the diffusion model learns the average distribution of real image data, since latent representations of synthetic data is enclosed by representations obtained for real image data. Sketch representations form a more distinct cluster, further promoting the realism of synthetic image data. (PDF) [file pcbi.1011890.s001.pdf]

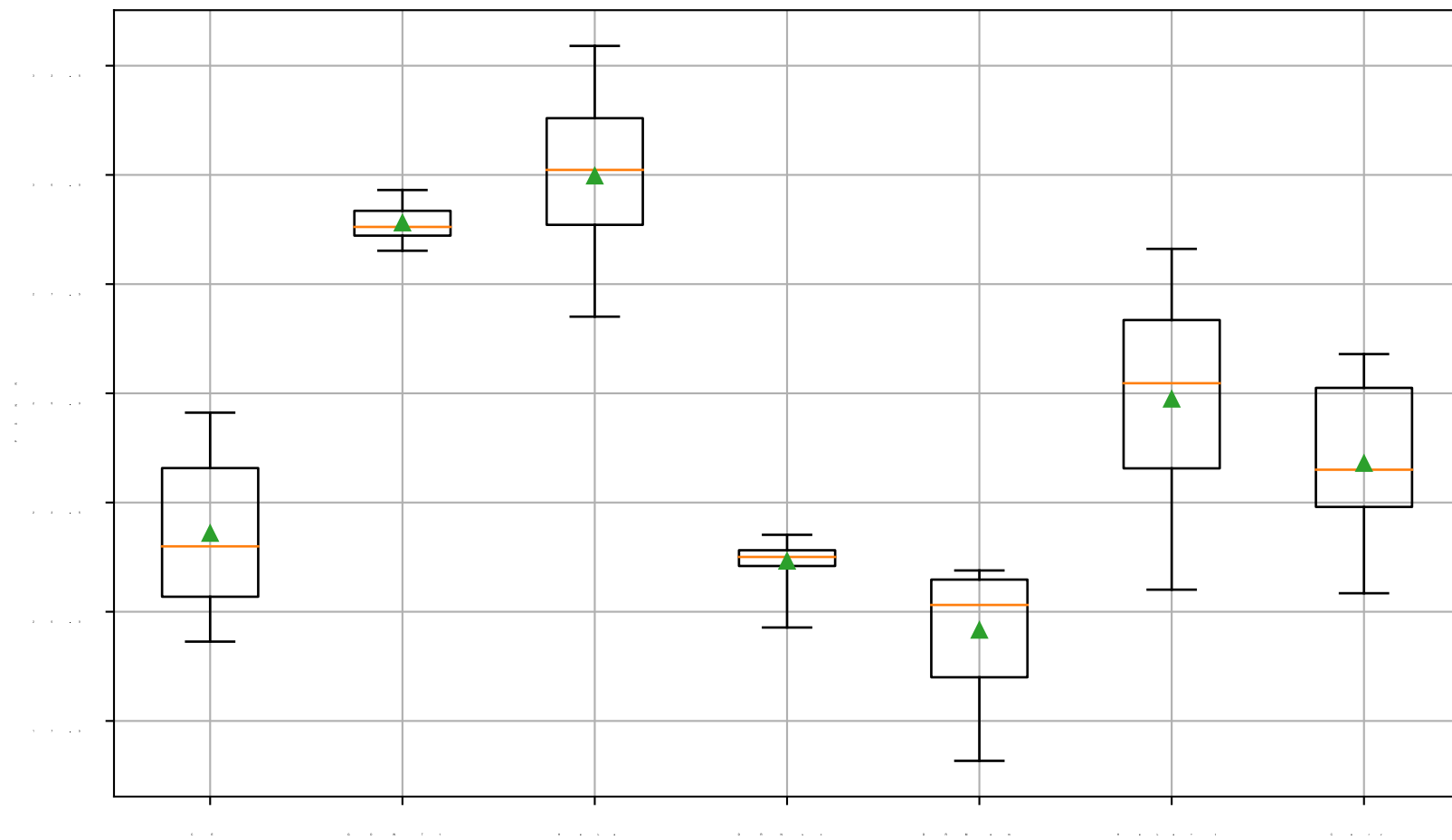

Supplement: S2 Fig — PSNR values presented as boxplots, calculated between real image data and synthetic image data generated from corresponding silver truth and segmentation masks. Those silver truth segmentations are generated with automated approaches and validated to be reliable for training purposes. Whiskers range from the 5th to the 95th quantile, median values are indicated as orange line while mean values are depicted as green triangle and boxes represent the interquartile range. The involved datasets are 3D Caenorhabditis elegans (CE) [19], 2D Mouse Stem Cells (GOWT1) [19], 2D HeLa Cells (HeLa) [19], 3D Nuclei and Membranes of Danio rerio (DRNuc,DRMem) [21], 2D+t mitotic progression in Mouse Stem Cells (HeLa+t) [24] and 2D overlapping cervical cancer cells (Cerv) [39]. Using corresponding sketches and the optimized settings of tstart = 400 and σ = 1, the backward process was used to replicate corresponding image samples and PSNR values were calculated to assess similarity between synthetic and real versions. The Danio rerio multi-channel data and the temporal HeLa data present special cases, which demonstrate limitations of the proposed approach. (PDF) [file pcbi.1011890.s002.pdf]

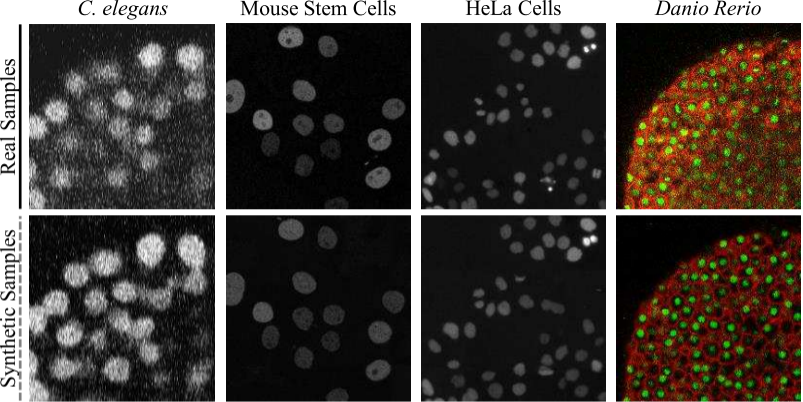

HeLa Cells (Mitotic Progression)

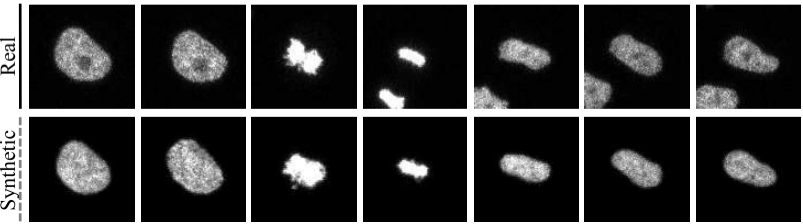

Supplement: S3 Fig — Examples of real image data and corresponding synthetic image data generated from corresponding silver truth and segmentation masks. The involved data sets are publicly available in [10, 19, 20, 22–24]. In case of the temporal HeLa data set, the 2D+t image stacks were treated as regular 3D images to allow for the generation of temporally consistent textures within the cellular and background regions. This was motivated by observations from previous experiments showing that a frame by frame generation of the temporal data is feasible but prone to the generation of slight textural inconsistencies that we were not yet able to prevent otherwise. (PDF) [file pcbi.1011890.s003.pdf]

Real Samples

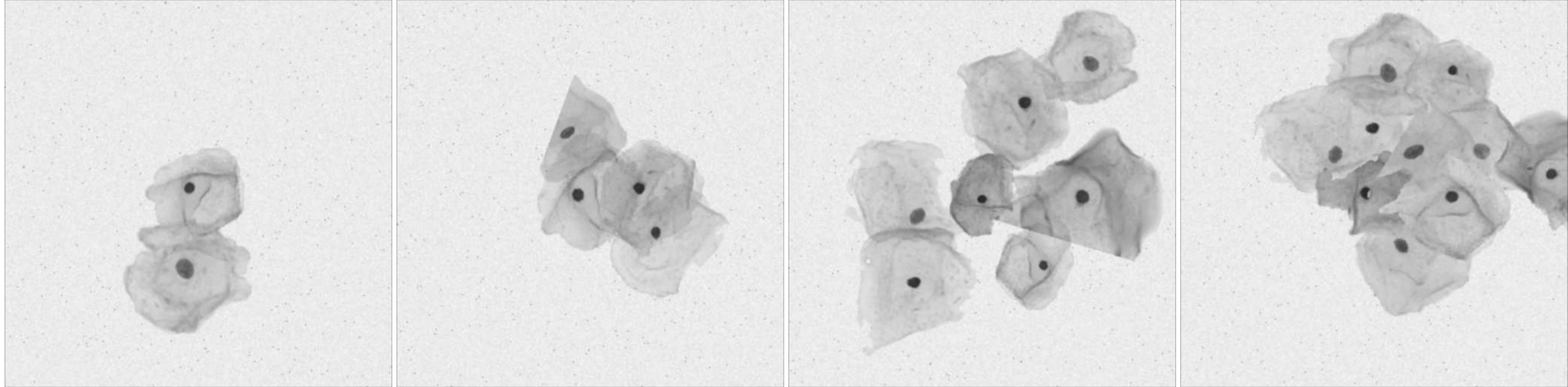

Synthetic Samples

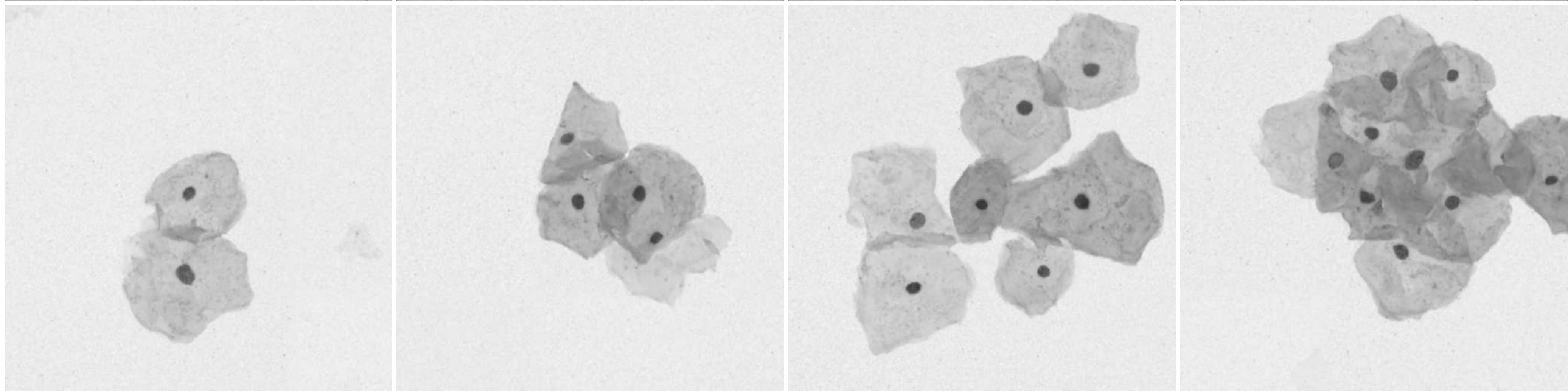

Real Samples

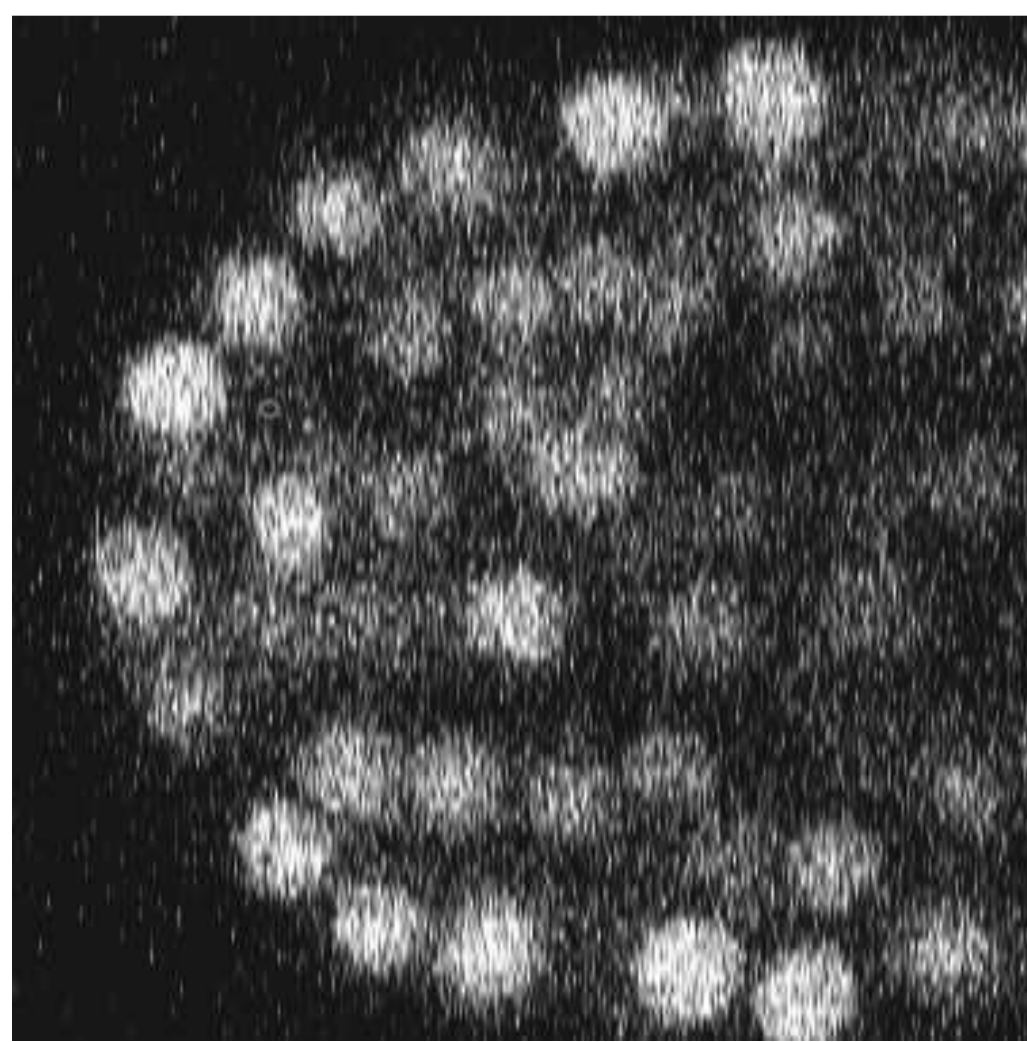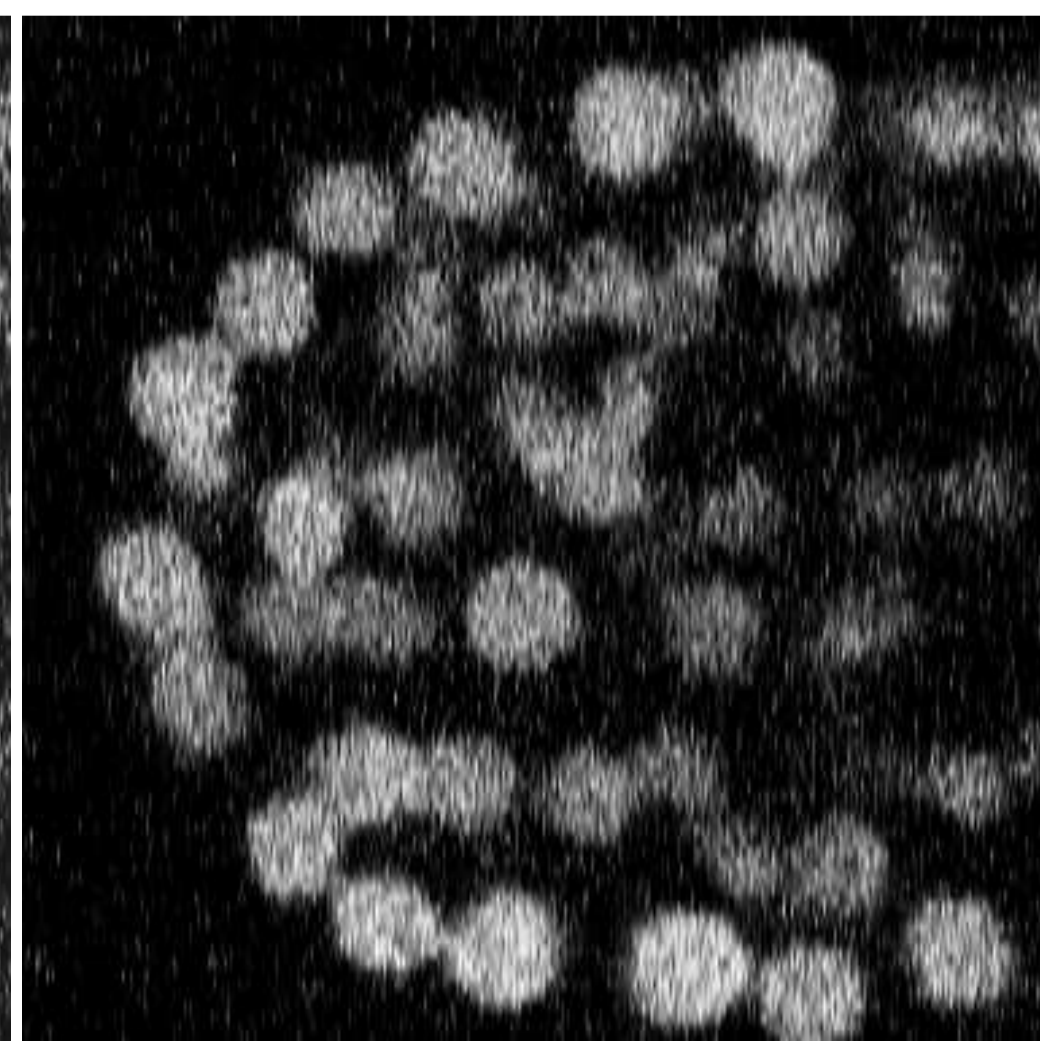

Synthetic Samples

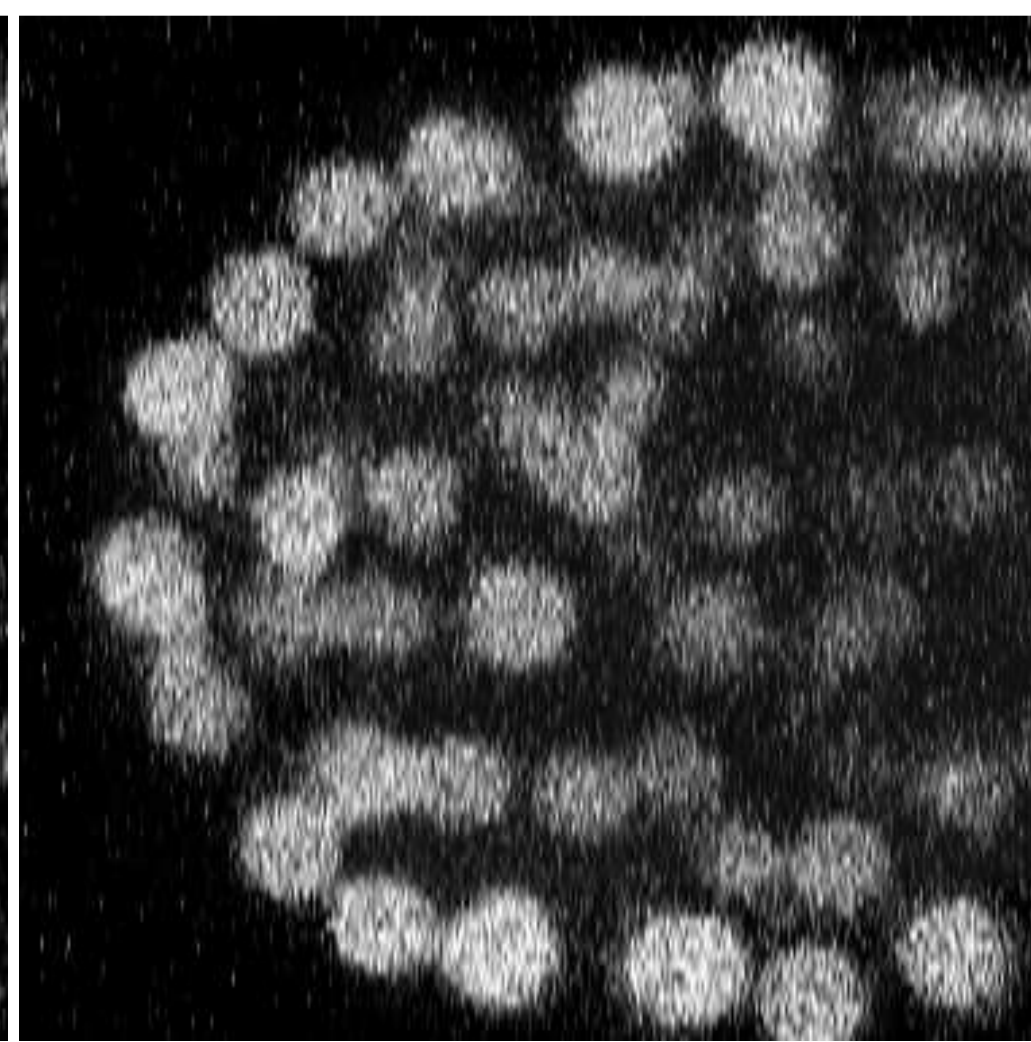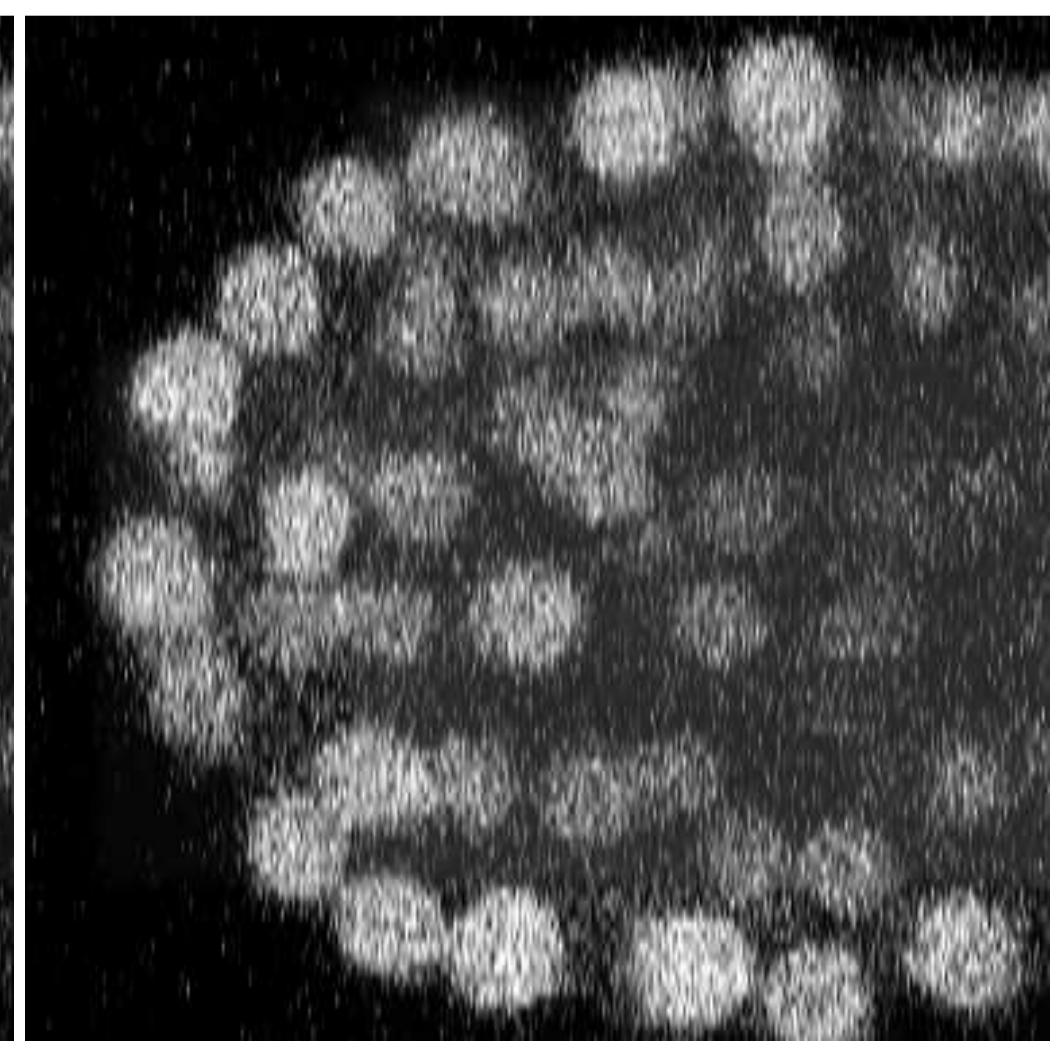

Supplement: S4 Fig — Examples of real image data and corresponding synthetic image data generated from corresponding annotations of overlapping cells [39] (top). Moreover, examples of varying background illumination in C. elegans [19, 20] was generated by adding indications within the sketches (bottom). This demonstrates the intuitive strength of the proposed approach, as more complex scenes of overlapping cells can be realistically generated and position-dependent texture characteristics can be straightforwardly imposed and controlled. (PDF) [file pcbi.1011890.s004.pdf]

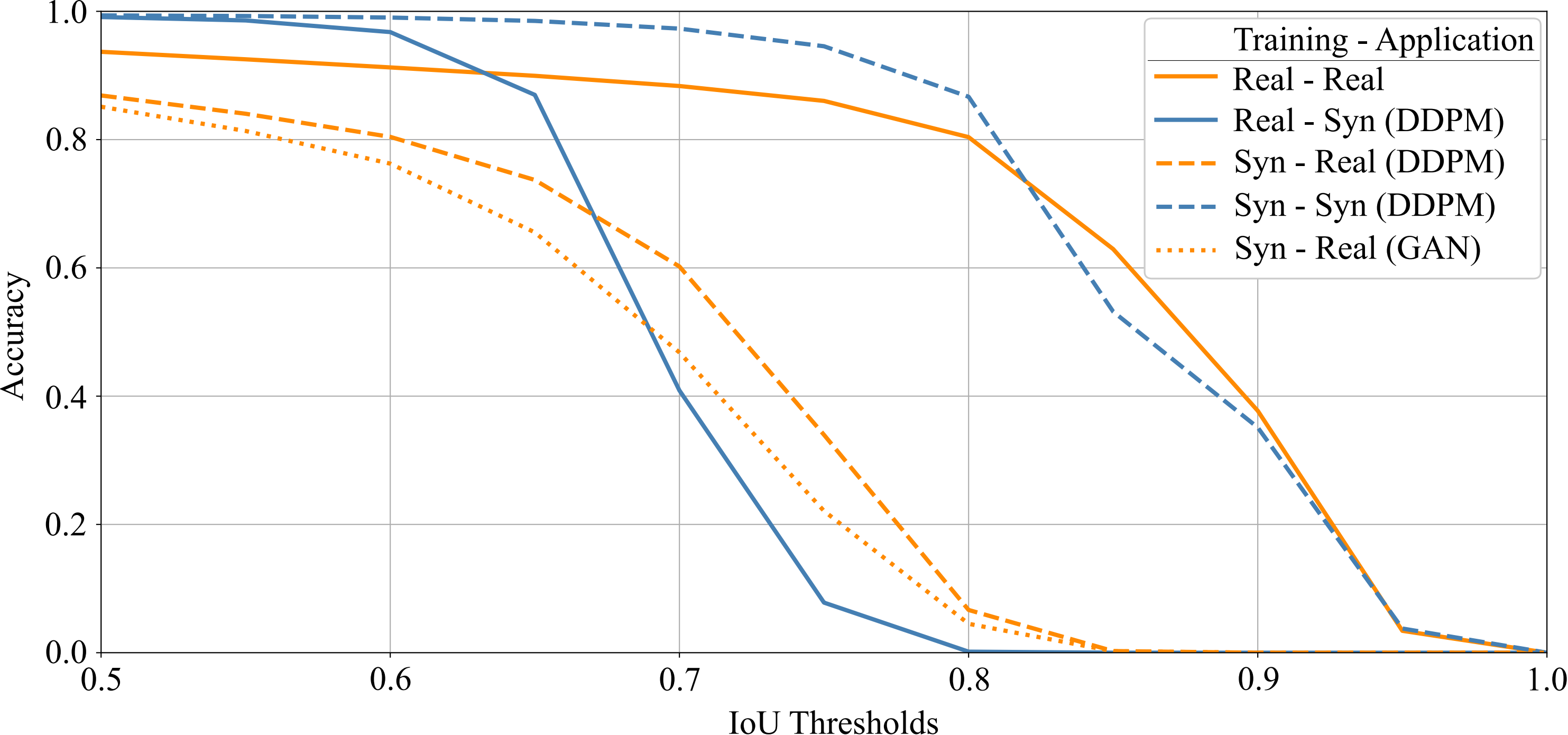

Supplement: S5 Fig — 3D Cellpose [27] segmentation models are trained on synthetic image data and real image data respectively, using the 3D Arabidopsis thaliana data set [18]. Both models are tested on real and synthetic image data. A model trained on GAN-generated image data is tested on real image data for a comparison between diffusion-based and GAN-based approaches. IoU scores are calculated for each cell in the image data and an IoU threshold provides the basis to formulate an accuracy as the ratio of precise segmentations to the total quantity of cells. It should be noted that no augmentation or dedicated pipeline tweaking was used during the training of the segmentation approaches, in order to obtain results that purely reflect the capabilities of the synthetic data. (PDF) [file pcbi.1011890.s005.pdf]

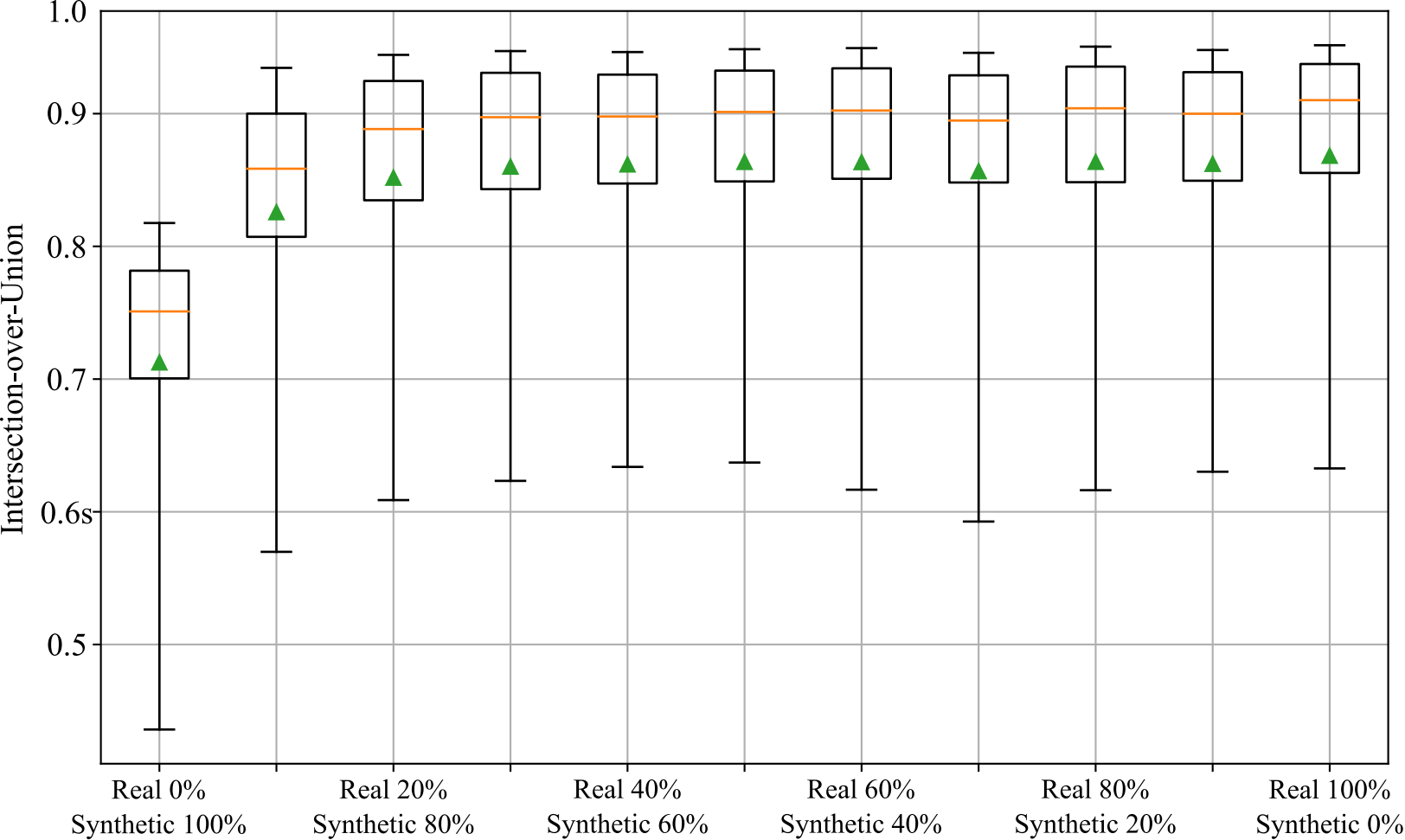

Supplement: S6 Fig — To present an analysis from a practical point of view, 3D Cellpose [27] segmentation models are trained on datasets containing a mix of synthetic and real image data from the 3D Arabidopsis thaliana data set [18]. The total quantity of training images was kept consistent throughout all experiments and all models are tested on real image data with the segmentation accuracy being reported by the IoU metric. Whiskers of the boxplots range from the 5th to the 95th quantile, median values are indicated as orange line while mean values are depicted as green triangle and boxes represent the interquartile range. (PDF) [file pcbi.1011890.s006.pdf]

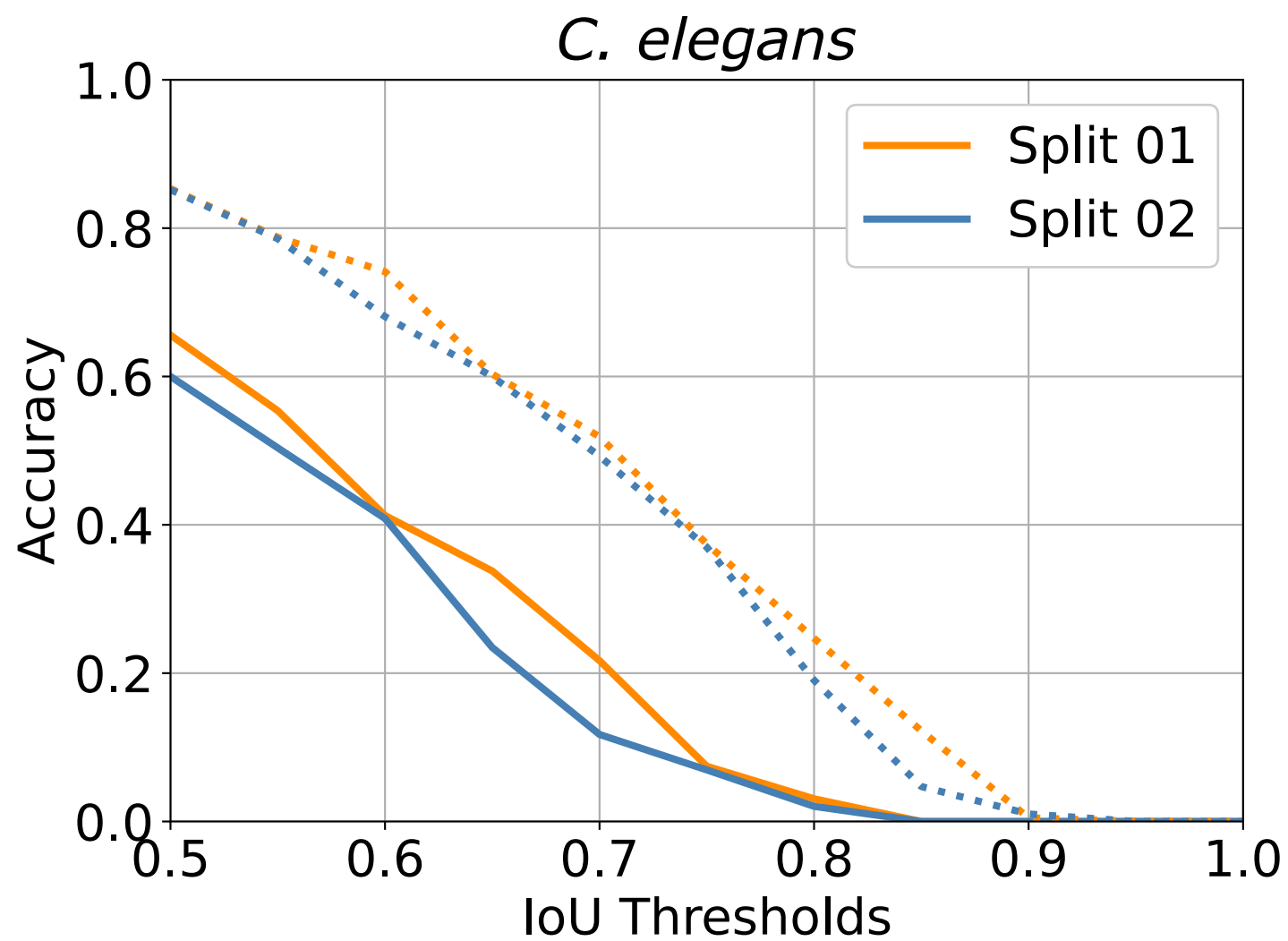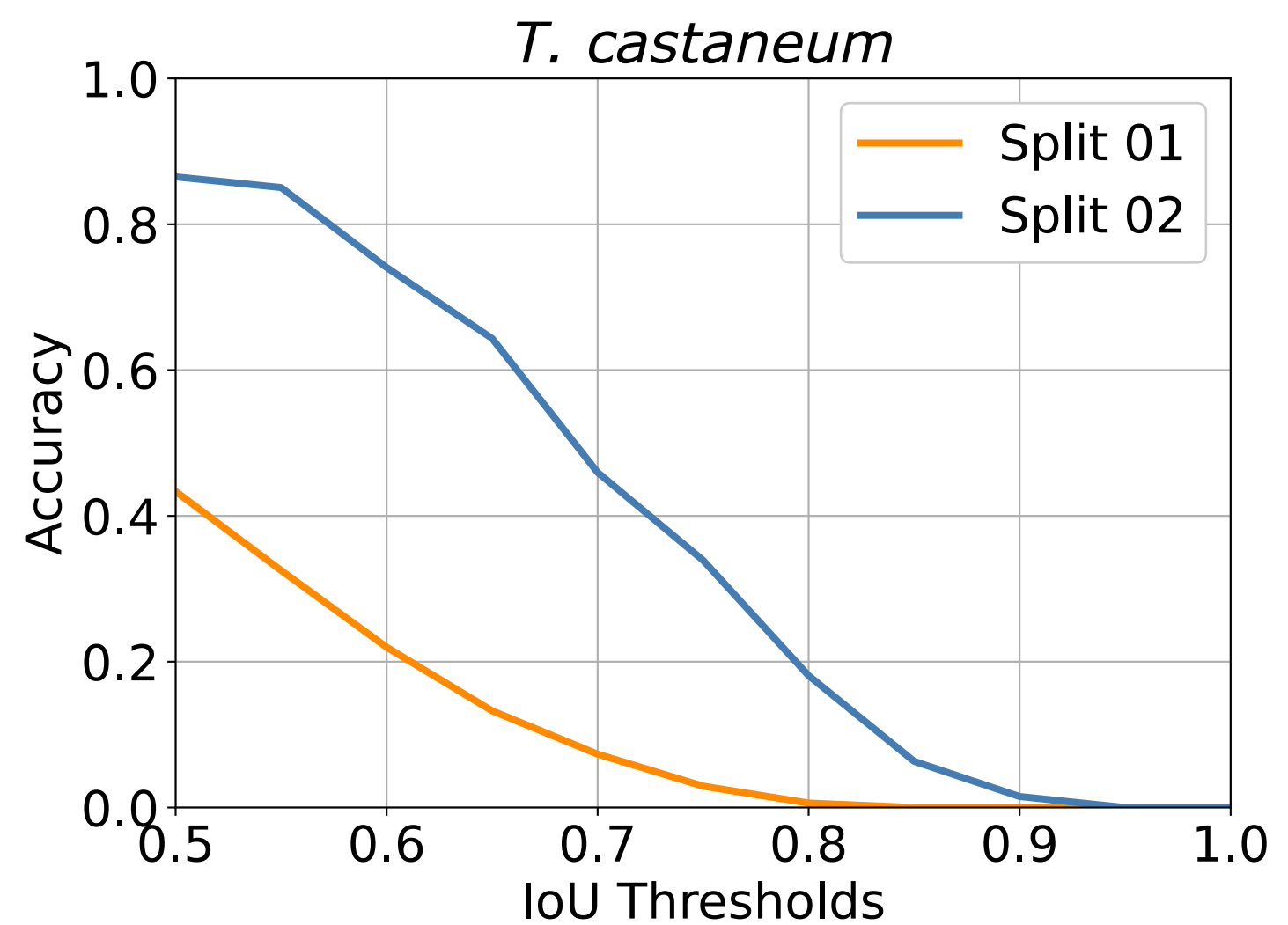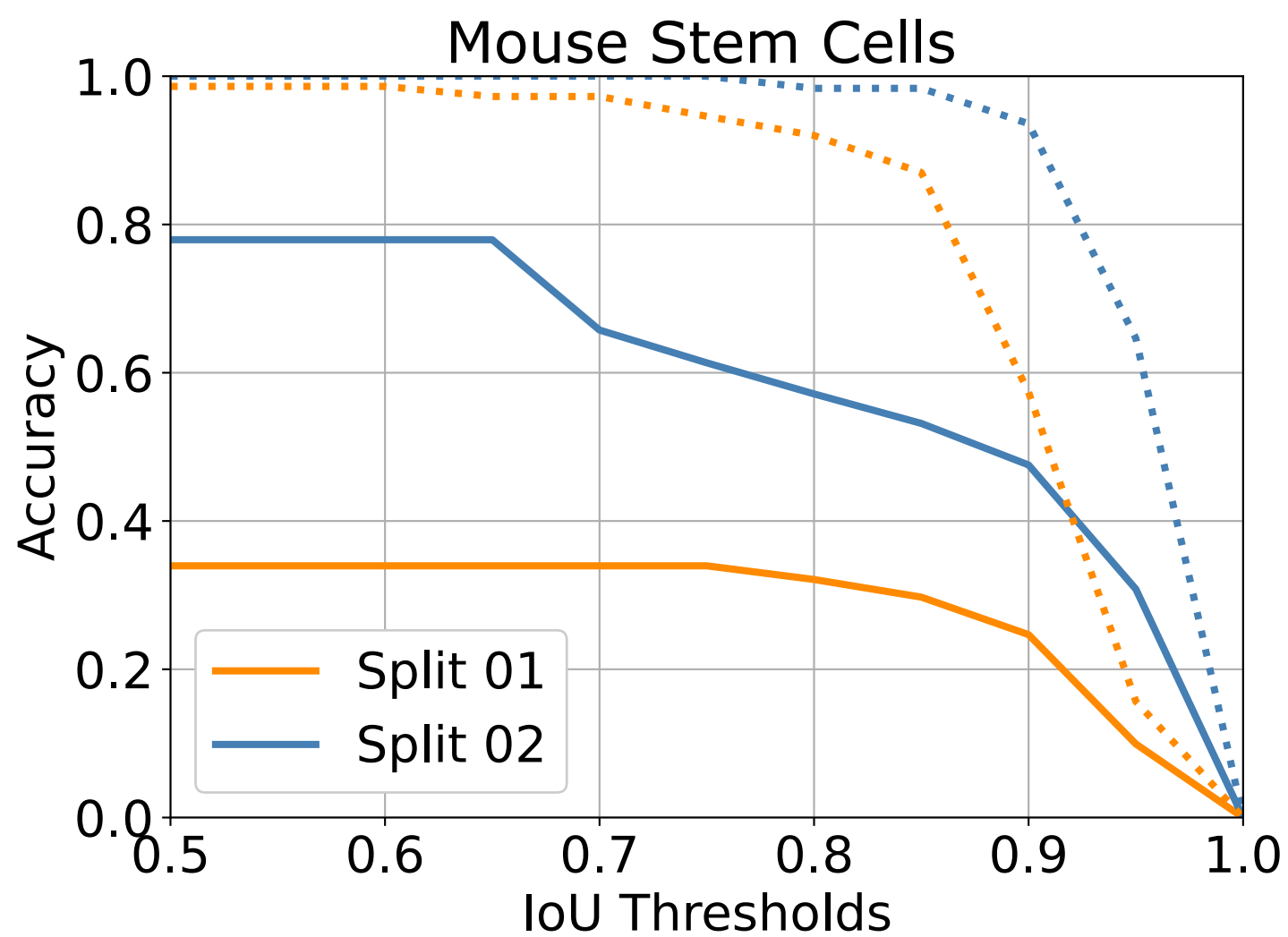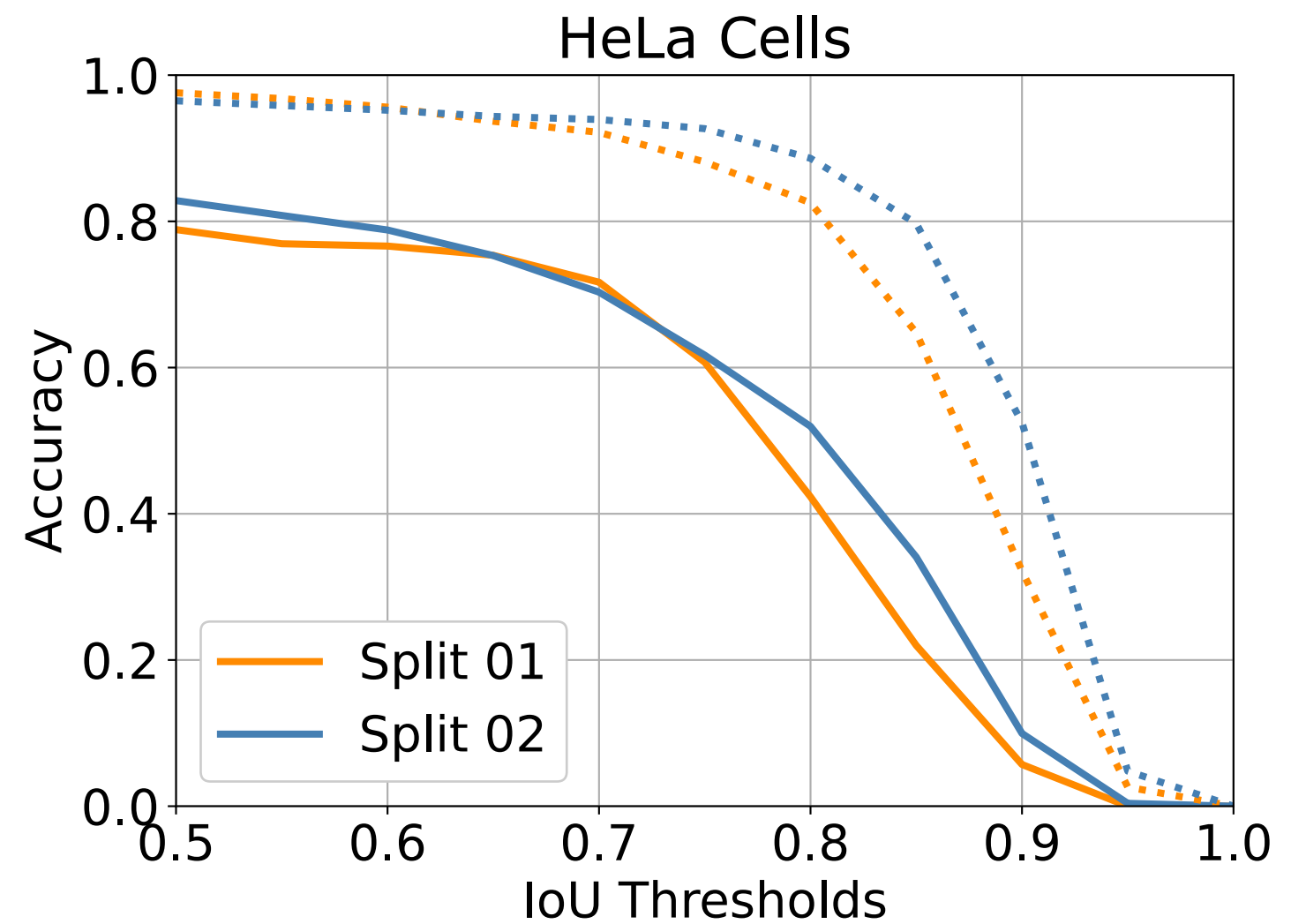

Supplement: S7 Fig — Solid lines show results obtained for segmentation models solely trained on synthetic data evaluated on the sparse manually annotated ground truth. Dotted lines show determined accuracies when considering the silver truth annotations as predictions (not provided for T. castaneum), and comparing results against the ground truth. Data splits, ground truth and silver truth were provided by the Cell Tracking Challenge [19]. Note that manual annotation are only provided for a small fraction of cells visible within the image data, and annotated cells often focus the most challenging regions, which are typically difficult to generate. (PDF) [file pcbi.1011890.s007.pdf]

Ground Truth

*C. elegans*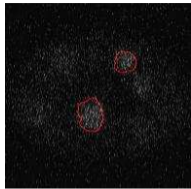*T. castaneum*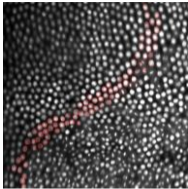

Mouse Stem Cells

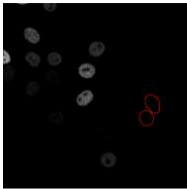

HeLa Cells

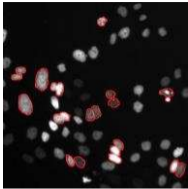

Segmentation

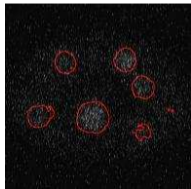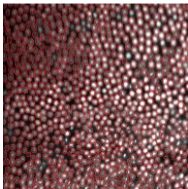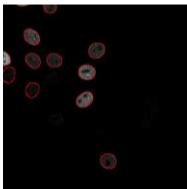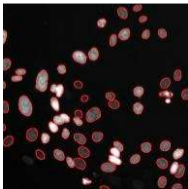

Supplement: S8 Fig — Examples of sparse ground truth annotations (top) and corresponding segmentations obtained by models solely trained on fully-synthetic image data generated from simulated sketches (bottom). The datasets and ground truth annotations are provided by the Cell Tracking Challenge [19]. Note that the annotations have never been used for training and are merely depicted for qualitative comparison. No data augmentation was applied. (PDF) [file pcbi.1011890.s008.pdf]
