# Supplementary material for: Denoising diffusion probabilistic models for generation of realistic fully-annotated microscopy image datasets
Source: PLoS Comput Biol. 2024 Feb 20;20(2):e1011890. doi: 10.1371/journal.pcbi.1011890 (PMC10906858; doi:10.1371/journal.pcbi.1011890)
Supplement: Attachment — Submitted filename: rebuttal_letter.pdf [file pcbi.1011890.s009.pdf]

Lehrstuhl für Bildverarbeitung
RWTH AACHEN UNIVERSITY

Prof. Dr.-Ing Johannes Stegmaier
Institute of Imaging and Computer Vision

Dennis Eschweiler, M.Sc.
Research Scientist

Room 107
Kopernikusstraße 16
52074 Aachen
Germany

Phone: +49 241 80-22917
dennis.eschweiler@lfb.rwth-aachen.de

05.01.2024

Dear editors and reviewers,

we would like to cordially thank all of you for your valuable suggestions and remarks. To meet the layout requirements, we restructured the chapters of the main script and the supporting material. Some parts of the supporting material were moved to the "Materials and methods" Section of the main script and all supporting Figures have received an improved caption and a corresponding reference Section in the main part. If there are more changes required, we are happy to further adapt the structure and content. In the following we would like to address all points raised by the reviewers, and hope we were able to resolve all concerns.

Reviewer #1

1. "The apparent requirement of the annotation be presented as a set of non-overlapping regions in a 2D image limits the ability to generate. As seen in Fig 2b or 3a, the real data close-up shows features of structures that exist in 3D that are partially overlapping in the 2D slice."
Unfortunately, we have to admit that we don't fully understand what is meant by those suggestions. If this addresses the lack of datasets showing overlapping cells, we would like to refer to our comment to point 3. In case this addresses the need to maintain the positions of structures throughout the data generation steps in order to have overlapping image/mask pairs as a result, we agree that this is an important factor that required optimization. This is primarily shown in Fig. 2b, but in our experiments this optimized setting proved to be robust as validated by all other experiments and no further tweaking was required. We would also like to point out that a large portion of the presented examples include 3D image data for which we trained corresponding 3D models. Even though the data was generated in a proper 3D fashion, the visualizations we provide only include 2D slices, which might cause some structures to appear overlapping.

2. "The diffusion model does not appear capable of generate more general artifacts over different regions of the image. This is most clearly seen in Fig 3a T. castaneum where the real dataset has different noise properties over different regions but not in the synthetic datasets."
In case of the *T. castaneum* data it is indeed true that the simulated structures show homogeneous illumination patterns, unlike the real image data. We account this to the design of the sketches, which were kept rather simple. However, the proposed pipeline itself has the capability to generate diverse scenes with more complex illumination characteristics. In order to demonstrate this, we set up a small experiment generating different background illuminations for the *C. elegans* dataset that we added to the supplementary material (Fig. S4). Here we chose the *C. elegans* dataset due to the apparent presence of a slightly illuminated organism region, which is easy to spot and was also neglected in the initial design of the corresponding sketches. We believe this experiment shows that local characteristics can be straightforwardly altered by providing appropriate indications within the sketches.

3. “The examples provided do not include examples of particularly challenging datasets, such as densely packed and labeled cells with close borders. The focus on labeled membranes or nuclei is understandable but it would be useful to better see limitations of the technique with cytoplasmic labeling.“

We agree that the choice of datasets was focused on fluorescence microscopy, mostly presenting non-overlapping cells. To this end, we added another experiment with a dataset showing cytology images of highly overlapping cancer cells (more details listed in Section S1). Training a model on the real images and utilizing the provided annotation masks of the test-split to create sketches, we generated corresponding synthetic image data (Fig. S4) that shows PSNR scores similar to the scores obtained for less complex scenes (Fig S2). This shows that the capabilities reach beyond the previously shown experiments, demonstrating the claimed flexibility and intuitiveness.

4. “It would be instructive to see synthetic datasets generated explicitly on 2D+t data and see if motion artifacts can be generated in data with moving cells.“

One of the listed experiments includes temporal 2D+t sequences of developing HeLa cells. Corresponding 2D+t synthetic data (Fig. S3) was generated by interpreting the whole 2D+t stack as a regular 3D image stack. We observed that this was necessary in order to generate temporally consistent noise and texture patterns, which we were not able to maintain otherwise. In our interpretation, all image characteristics could be realistically recreated, although we acknowledge that the necessity to generate the entire pseudo-3D stack to reach realism still poses a limitation. Finding solutions to overcome this limitation is already part of our future work.

Reviewer #2

1. “It is shown for a given domain that training on synthetic data and applying to real data leads to results comparable to the generalist cellpose model (which was trained on a variety of different domains and is actually a 2D model). To actually demonstrate the claimed main utility of the approach (elevating the annotation burden), however, one would need to compare it to a model that was solely trained on the real annotations from the *same* domain. The supplement figure 4 seems to show the results of that experiment, but the legend is unclear (Is this "SynDiff2Real" vs "Real2Real"?). If so, the reduced accuracy (~ 0.87 for synth vs ~0.95 for real for thresh 0.5) actually would show that there is still quite a large drop potentially due to the domain gap? I think adding these numbers (for the real domain specific 3D cellpose model) to the main Fig3 is merited. Related to that, it would be important from a practical point of view to know at which amount of real annotation masks this real domain specific model surpassing the one trained on synthetic data. This should be a fairly simple experiment and would inform the reader what order of magnitude of real annotations would be needed to "catch up" with the (cheap) synthetic annotations.“

Figure S5 (previously S4) indeed shows the results for the mentioned experiment. Here we trained two models of the 3D Cellpose extension, one using real image data including manual annotations and the other one using synthetic image data based on simulated sketches/annotations. For better clarity we changed the description of the different models and an explanation of this experiment to the main part of the paper in order to emphasize the obtained outcomes and the presented domain gap, as suggested. The gap is indeed still present and leads to a drop in segmentation accuracy, even though results could be improved compared to the GAN-based data generation. However, we want to emphasize that the reported scores can still be maintained on a high level. Adding to this, we set up the requested experiment to evaluate how various combinations of real and synthetic data samples affect the outcome (Fig. S6). We found that the accuracy of the segmentation model solely trained on manual annotations can be reached by substituting 70-80% with synthetic samples.

2. “The mask/sketch creation process seems to be very intricate and domain specific. First, one needs a domain specific generative model of the mask (e.g. nuclei) distribution and then another for the domain specific mask->sketch generation (e.g. decreasing intensity along the z axis for A. thalania or the nucleoli placement for Mouse stem cells). It thus seems that the needed amount of knowledge how to design this pipeline for a new (and potentially more complicated) domain might be overly challenging thus defeating the premise of the paper. Is there a way to assess how important the used pipeline details are for the final segmentation results? (e.g. does it drop considerable when not using the axial intensity profile and the shape model of S[3] or is it actually quite robust to it?)“
We agree that the shift from requiring manual annotations to requiring appropriate structure simulation techniques also comes with a cost. However, we want to emphasize that this shift provides the means for higher re-usability and sustainability. Simulation techniques and model knowledge, once acquired, can be applied across multiple biomedical experiments, where the diffusion model would solely require training on real image data to comprehend noise and texture characteristics. All of this would come without extra costs and could be performed automatically. The potential requirement to add additional rough indications to sketches is typically a straightforward task that does not pose any challenge as shown in our experiments. Conversely, utilizing manual annotations necessitates acquiring additional annotations for any new noise and texture features, resulting in added costs. Moreover, we want to point out that the utilized simulation techniques are already kept rather simple, e.g., only utilizing ellipsoids and deformation fields to generate all nuclei shapes, which we believe is applicable to a large variety of other datasets. Nevertheless, we must agree that setting up these simulation techniques is the main challenge for obtaining realistic outcomes. On this matter, we conducted additional experiments where we tried to generate synthetic image data for the, arguably most complex, *A. thaliana* dataset by only creating patches of simple sketches using straightforward Delaunay triangulations, neglecting the organism outline/shape. However, using those sketches we were not yet able to reach the segmentation accuracy we obtained when using more complex whole organism simulations as a basis, indicating that accurate local characteristics are actually important. This is still part of our current research, since we believe it should be possible to utilize less complex patch-based simulation techniques, when using segmentation models that are trained and applied in a patch-based manner anyways.

Minor Points:

“What are the memory requirements esp. for the 3D image generation? What image size is feasible to generate?”
“Could you add the rough training times/GPU specs needed for the 2D and 3D DDPM?”
Due to the structure and working principle of our computation cluster that houses a variety of different hardware capabilities, both of these details were difficult to accurately determine. That’s why we thought adding this information to the paper would lack reliability. Nevertheless, we agree that hardware requirements are important to know from an application point of view and added some indications to our repository. We also want to point out that the processing is done in a patch-based manner, which eases memory constraints.

“The way how to use the code on new datasets is currently hard to understand from the github repo description. (How to convert the images? How to adapt the parameters, typos like "utils.h5_conveter.prepapre_masks"). I would suggest to provide example images/masks to download together with the concrete script calls to run the training/prediction pipeline on those.”
We agree that the previous instructions were lacking some details and, therefore, added further information to the repository. This includes an additional Jupyter notebook introducing the data preparation steps and, as suggested, an image/mask pair that can be used with all provided notebooks

for preparation, training and prediction. Furthermore, we added a pretrained model to the repository, such that the prediction pipeline can also be tested without the need to make further adjustments. We hope this helps to ease the application and provides more clarity.
